# The complete mitochondrial genome sequence and phylogenetic analysis of *Cantharis plagiata* (Coleoptera, Canthridae)

**DOI:** 10.1080/23802359.2020.1775515

**Published:** 2020-06-08

**Authors:** Hua-Cong Xi, Shu-Juan Ge, Zi-Xuan Kang, Hao-Yu Liu, Yu-Xia Yang

**Affiliations:** The Key Laboratory of Zoological Systematics and Application, College of Life Sciences, Institute of Life Science and Green Development, Hebei University, Baoding, Hebei Province, China

**Keywords:** Mitochondrial genome, Cantharis plagiata, Cantharidae

## Abstract

The complete mitochondrial genome of a soldier beetle, *Cantharis plagiata* (Coleoptera, Canthridae), was sequenced. The mitogenome is a double-stranded circular molecule, and the obtained sequence had 13 protein-coding genes (PCGs), 22 tRNA genes, 2 rRNA subunits, and an AT-rich region, as in other insects. Total length of this mitogenome is 16,315 bp and the composition of each base is A (41.4%), T (37.5%), C (12.7%), G (8.4%), respectively. The phylogenetic tree analysis using 25 species of Elateroidea showed that *C. plagiata* is closest to *C*. *pellucida*, which confirms its systematic status in Cantharidae.

*Cantharis* (*Cyrtomoptila*) *plagiata* Heyden, 1889 is a species belonging to the family Cantharidae. The species is small-sized, about 5.5–7.0 mm in length, and it could be easily recognized by the light yellow body coloration, except the orange pronotum, which has a black longitudinal band along the middle line; all outer claws are toothed in male, while simple in female (Yang and Yang 2018).

*Cantharis plagiata* is widely distributed in Far East Russia, Korea, Japan and China (Kazantsev and Branucci 2007), within which it spreads from the northern to central and western parts, including Heilongjiang, Hebei, Beijing, Tianjin, Shanxi, Shaanxi, Gansu, Hubei and Sichuan (Yang 2010).

The specimens used in this study were collected from Foyedong, 963.6 m, 33.45°N, 107.36°E, Yangxian, Shaanxi Province, China, and stored in the Museum of Hebei University, Baoding, China (MHBU), with an accession number CAN0074. Genomic DNA was extracted by DNeasy Blood & Tissue kit (QIAGEN, Germany). Illumina TruSeq libraries were prepared using genomic DNA with an average insert size of 450 bp and were sequenced on the Illumina Hiseq2500 platform with 250 bp paired-end reads at BerryGenomics (Beijing, China). The sequence reads were first filtered by the programs following Zhou et al. (2013) and then the remaining high-quality reads were assembled using IDBA-UD (Yu and Henry 2012). In order to study the accuracy of assembly, Geneious 2019.2 was used to map clean reading onto the mt genome sequence. The annotations of genes were done by Geneious 2019.2 software and tRNAscan-SE 1.21 (Schattner et al. 2005). Annotated sequence was registered in the GenBank with an accession number MT364421.

The complete mitochondrial genome (mitogenome) of *C. plagiata* is a double-stranded circular molecule of 16315 bp in length, which contains 22 tRNA genes, 13 protein-coding genes (PCGs), 2 rRNA genes, and an AT-rich region as in other insects. The composition of each base was calculated as A (41.4%), T (37.5%), C (12.7%), G (8.4%), and GC content was 21.1% with a much higher AT content. The start codons of 13 PCGs were all ATN. In addition, an incomplete terminal codon AA was found in COI. In case of other 12 CDSs, TAA or TAG was used. The length of the AT-rich region was 1432 bp.

The neighbour-joining tree was constructed by MEGA 7.0 with 1500 bootstrap replicates, based on Kimure-2 parameter model using 25 species of Elateroidea as inner group and 1 species of Dryopidae, Scirtidae and Dascillidae, respectively, as outer group. These species were as follows: *Drilaster* sp. (Timmermans et al. 2010), *Bicellonychia lividipennis* (Amaral et al. 2016), *Photinus pyralis* (Fallon et al. 2018), *Lampyris noctiluca* (Linard et al. 2018), *Luciola cruciata* (unpublished), *Agriotes obscures* (Linard et al. 2016), *Melanotus villosus* (Linard et al. 2016), *Anostirus castaneus* (Linard et al. 2018), *Limonius californicus* (Gerritsen et al. 2016), *Drilus flavescens* (Timmermans et al. 2010), *Teslasena femoralis* (Amaral et al. 2016), *Dicronychus cinereus* (Linard et al. 2018), *Hapsodrilus ignifer* (Amaral et al. 2016), *Lycus dentipes* (Timmermans et al. 2010), *Platerodrilus* sp. (Uribe et al. 2016), *Lycostomus* sp. (Liu et al. 2019), *Chauliognathous opacus* (Sheffield et al. 2009), *Cantharis pellucida* (Sheffield et al. 2009), *C*. *plagiata* (this study), *Rhagonphthalmus lufengensis* (Li et al. 2007), *Brasilocerus* sp. (Amaral et al. 2016), *Melasis buprestoides* (Timmermans et al. 2010), Cerophytidae sp. (unpublished), *Iberobaenia minuta* (Andújar et al. 2017), *Omalisus* fontisbellaquei (Timmermans et al. 2015), *Elodes mimuta* (Linard et al. 2018), *Dryops ernesti* (Linard et al. 2018) and *Dascillus cervinus* (Timmermans and Vogler 2012). The phylogenetic inference was done based on 13PCGs. Trans Align methods were used to align all protein-coding genes (Bininda-Emonds 2005). The aligned data from 13PCGs were concatenated with Sequence Matrix v.1.7.8 (Vaidya et al. 2011). Data were partitioned according to loci of 13 PCGs. The bootstrap showed sufficient value at all nodes. The result (Figure 1) showed that *C. plagiata* was definitely closest to *C*. *pellucida* with 100% bootstrap value, and the two species were further grouped with *Chauliognathus opacus*. All three species belong to the family Cantharidae, the former two are placed in the subfamily Cantharinae and the latter in Chauliognathinae.

## Data Availability

The data that support the findings of this study are openly available in GenBank of NCBI at http://www.ncbi.nlm.nih.gov, reference number MT364421. The phylogenetic tree of 25 species of Elateroideabased on 13 PCGs of mitochondrial genome sequence.
